# MMP-9 Activation via ROS/NF-κB Signaling in Colorectal Cancer Progression: Molecular Insights and Prognostic–Therapeutic Perspectives

**DOI:** 10.3390/cimb47070557

**Published:** 2025-07-17

**Authors:** Andrej Veljkovic, Goran Stanojevic, Branko Brankovic, Stefanos Roumeliotis, Konstantinos Leivaditis, Branka Djordjevic, Xiaobo Li, Aleksandra Klisic, Jovan Hadzi-Djokic, Gordana Kocic

**Affiliations:** 1 Faculty of Medicine, University of Nis, 18000 Nis, Serbia; brankadjordjevic83@gmail.com; 2 Clinical Center, 18000 Nis, Serbia; goranstanojevic1010@gmail.com (G.S.); branko.brankovic@medfak.ni.ac.rs (B.B.); 3 2nd Department of Nephrology, AHEPA Hospital, School of Medicine, Aristotle University of Thessaloniki, 541 24 Thessaloniki, Greece; st_roumeliotis@hotmail.com (S.R.); konleiv@windowslive.com (K.L.); 4 Department of Pathology, Harbin Medical University, Harbin 150081, China; lixiaobo@ems.hrbmu.edu.cn; 5 Department of Biochemistry, Faculty of Medicine, University of Montenegro, 81000 Podgorica, Montenegro; 6 Center for Laboratory Diagnostics, Primary Health Care Center, 81000 Podgorica, Montenegro; 7 Serbian Academy of Sciences and Arts, 11000 Beograd, Serbia; jovanhdj@gmail.com (J.H.-D.); kocicrg@yahoo.co.uk (G.K.)

**Keywords:** colorectal cancer, NF-κB, AOPP, MMP-9

## Abstract

Colorectal cancer (CRC) is characterized by complex interactions between inflammation, oxidative stress, and extracellular matrix remodeling. Recent studies have highlighted the significance of the reactive oxygen species (ROS)–nuclear factor kappa B (NF-κB)–matrix metalloproteinase-9 (MMP-9) axis in promoting tumor invasion and metastasis in CRC, linking oxidative stress with inflammatory signaling and extracellular matrix degradation. In this study, we analyzed the concentration of advanced oxidation protein products (AOPPs), expression of NF-κB, and the activity of MMP-9 in tumor tissue, adjacent tissue, and healthy control colon tissue. Tissue specimens were collected from 50 patients with primary CRC following surgical resection. The analyses were performed using appropriate and validated biochemical methods, including ELISA, spectrophotometry, and indirect immunofluorescence. Significantly higher levels of all three markers were observed in tumor tissue compared to controls. Additionally, adjacent tissue exhibited elevated NF-κB expression and MMP-9 activity when compared to healthy colon tissue. AOPP levels correlated strongly with MMP-9 activity, highlighting the role of oxidative stress in the activation of MMP-9. MMP-9 demonstrated the highest predictive value for CRC, emphasizing its potential as a diagnostic and theranostic marker. Our findings support the hypothesis that the ROS–NF-κB–MMP-9 axis plays an important role in CRC progression, particularly during stages T2 and T3. Targeting this pathway may offer new therapeutic strategies for limiting tumor invasion and recurrence. Moreover, ensuring adequate surgical resection margins is crucial to optimizing treatment outcomes.

## 1. Introduction

Colorectal cancer (CRC) is one of the most common neoplastic diseases in the human population and ranks as the third most prevalent malignancy worldwide. The incidence of CRC is rising rapidly, particularly in developing countries [1]. Over the past decade, changes in dietary patterns, especially in industrialized nations, have contributed to the increasing morbidity and mortality rates associated with CRC. This trend may also be partially attributed to improved diagnostic capabilities in countries with advanced healthcare systems. However, the precise mechanisms underlying the initiation and progression of CRC remain incompletely understood, warranting continued scientific investigation into its molecular pathogenesis.

One of the major challenges in CRC management is the local invasion of malignant cells into healthy colonic tissue. This, along with vascular infiltration, facilitates the metastatic potential of the tumor. Additionally, chronic low-grade inflammation is a well-recognized factor in various gastrointestinal disorders and plays a critical role in the development of malignancies, including CRC. Inflammation-driven carcinogenesis has been implicated in several tumor types, emphasizing the need for further research into the inflammatory mechanisms contributing to CRC progression [2,3].

The nuclear transcription factor κB (NF-κB) is a redox-sensitive regulator that plays a crucial role in the cellular response to stress signals. It is a key mediator of the inflammatory cascade and is strongly implicated in carcinogenesis [4,5]. By receiving signals from both the tumor and its microenvironment, NF-κB can be activated by various stimuli, promoting cancer cell survival, proliferation, invasion, and metastasis [5,6]. The activation of NF-κB represents a double-edged sword—while essential for normal immune function, its dysregulated activation can drive chronic inflammation and contribute to malignant transformation. This duality is particularly evident in cancer, which is widely recognized as a pro-inflammatory disease [7]. Activation of NF-κB is initiated by signal-induced degradation of IκB proteins. Upon stimulation, IκBα undergoes phosphorylation and ubiquitination-dependent degradation via the activation of the IκB kinase (IKK) complex [8,9]. The degradation of IκBα allows NF-κB to translocate into the nucleus, where it functions as a transcription factor by binding to specific DNA sequences and regulating gene expression [10]. It has been reported that chemotherapeutic agents can stimulate NF-κB activation, thereby enhancing chemoresistance mechanisms in cancer cells [11,12]. Since NF-κB is a redox-sensitive transcription factor, one of its activators includes reactive oxygen species (ROS), which are known to play a significant role in cancer initiation and progression [13]. Excessive ROS production has been observed in chronic inflammatory diseases of the digestive tract [14]. One of the oxidative stress markers resulting from ROS-induced protein damage is advanced oxidation protein products (AOPPs), which serve as indicators of oxidative injury. With the emergence of the “seed and soil” hypothesis, the influence of the tumor microenvironment on neoplastic cells has gained increasing attention, further emphasizing the interplay between oxidative stress, inflammation, and cancer progression [15].

During tumorigenesis, the extracellular matrix (ECM) plays a dual role—acting both as a structural barrier and as a facilitator of tumor growth, proliferation, and invasion [16,17]. This process begins with the adhesion of cancer cells to the ECM, followed by its degradation via the action of proteolytic enzymes [16]. One of the key proteases involved in ECM degradation is matrix metalloproteinases, making them crucial mediators of tumor growth, invasion, and metastasis [18,19]. Among them, MMP-9, also known as gelatinase B or 92-kDa type IV collagenase [20], plays a pivotal role in tumor invasion and angiogenesis by mediating the degradation of vascular structures that support tumor growth [21]. Overexpression of MMP-9 has been implicated as a key event in the multi-step process leading to neoplastic cell proliferation and metastasis [22]. However, the relationship between ROS, NF-κB activation at different stages of CRC, and ECM alterations due to MMP-9 activity remains insufficiently understood. The enzymatic degradation of colorectal mucosa in comparison to adjacent tissue and healthy colon tissue has not been thoroughly investigated in colorectal neoplasia.

To address these gaps in knowledge, this study aimed to assess changes in the quantitative expression of NF-κB, concentration of AOPPs, and MMP-9 activity in colon cancer tissue and adjacent tissue compared to healthy control tissue. This signaling pathway may serve as a potential marker for progression of the cancer. Furthermore, the study seeks to elucidate the molecular mechanisms linking oxidative stress, inflammation, and extracellular matrix remodeling through interactions between AOPPs, NF-κB, and MMP-9, with the ultimate goal of determining whether these parameters have prognostic significance and potential therapeutic relevance.

## 2. Materials and Methods

All the reagents were purchased from Sigma (St. Louis, MO, USA).

### 2.1. Patients and Tissue Samples

We conducted a study on 50 patients (median age: 56.4 years; 29 men and 21 women) diagnosed with CRC at the Clinical Center in Niš, Serbia. The study was conducted in accordance with the Declaration of Helsinki, and the protocol was approved by the Ethics Committee of the Faculty of Medicine in Niš (Decision No. 01-1591/8). Tissue specimens were collected from 50 patients with primary CRC, encompassing all four TNM clinical stages of the disease. Samples were obtained during surgery as soon as possible after tumor resection. For each patient, a tumor tissue sample was collected, followed by a sample of adjacent macroscopically normal tissue. The adjacent tissue was defined as macroscopically normal mucosa located closest to the tumor margin, and was confirmed histologically as tumor-free. As a control, a sample of the most distant healthy colon tissue was obtained. Healthy tissue macroscopically appeared normal, and confirmed by the pathologist as histologically without signs of malignancy or dysplasia. In all patients, pathological analysis confirmed the presence of adenocarcinoma. Patients with other histological types of tumors were excluded from the study. Additional exclusion criteria included pregnancy, other malignancies, inoperability, previous chemotherapy, or prior radiation therapy. Exclusion criteria also included presence of inflammatory bowel disease, autoimmune disorders, chronic kidney or liver disease, and recent antioxidant supplementation (within 3 months). These criteria aimed to minimize systemic inflammation or oxidative imbalance unrelated to the tumor.

### 2.2. Preparation of Tissue Samples

Tissue samples were rapidly collected during surgery. All samples were placed in ice-cold 0.15 mol/L NaCl solution and perfused with the same solution to remove blood cells and other tissue residues. Subsequently, the samples were blotted on filter paper, weighed, and homogenized under standardized conditions. Ten percent (*w*/*v*) homogenates were prepared, frozen at −20 °C, and stored until further analysis.

### 2.3. Biochemical Assays

#### 2.3.1. Quantitative Expression of NF-κB

The quantitative expression of NF-κB was determined by the indirect immunofluorescence method, following the protocol described by Hafiz A. [23], with modifications as previously reported in our study [24]. This method represents a standard enzyme-linked immunosorbent assay (ELISA). The mean fluorescence intensity was measured and analyzed using a Victor™ multiplate reader (PerkinElmer-Wallace, Wellesley, MA, USA). Results are presented as percentage changes relative to healthy colon tissue. Each sample had its own control (tissue treated with secondary antibodies only), and the fluorescence values of the controls were subtracted from the final analysis. The data were normalized to protein content and expressed per mg of protein.

#### 2.3.2. AOPP Concentration

The concentration of AOPPs was determined spectrophotometrically using the method described by Witko-Sarsat et al. [25]. A 200-μL aliquot of supernatant was diluted 1:5 in PBS, and chloramine-T standard solutions were prepared in a 96-well microtiter plate. This was followed by the addition of 20 μL of acetic acid and 10 μL of 1.16 M potassium iodide, after which another 20 μL of acetic acid was added. The absorbance of the reaction mixture was immediately measured at 340 nm using a microplate reader. AOPP concentration was expressed in μmol/mg chloramine-T equivalents.

#### 2.3.3. MMP-9 Activity

MMP-9 activity was determined using an ELISA-based commercial kit (SensoLyte Plus™ 520 MMP-9 assay system, AnaSpec, San Jose, CA, USA). A specific anti-MMP-9 monoclonal antibody was used in combination with a fluorogenic MMP substrate, 5-FAM/QXL^®^520 FRET peptide. The fluorescence signal was monitored at Ex/Em = 490 nm/520 nm upon MMP-9-induced cleavage of the FRET substrate. Enzyme activity was quantified using a standard curve and expressed as ng/mg of protein.

#### 2.3.4. Protein Content

The total protein concentration in tissue homogenates was determined using the method previously described by Popović et al. [26]. The reaction mixture contained 10 μL of the diluted tissue homogenate and 150 μL of the reagent C (1 mL of 1% CuSO_4_, 1 mL of 2% potassium sodium tartrate, and 98 mL Na_2_CO_3_ dissolved in 0.1 M NaOH). After 30 min of incubation, 30 μL of the Folin reagent was added to the mixture. After 20 min, the absorbance was read at 550 nm. Protein content was expressed as mg of protein/L.

### 2.4. Statistical Analysis

All data were tested for normality with the Kolmogorov–Smirnov test. We expressed non-normally distributed continuous variables as median with interquartile range and normally distributed continuous variables as mean ± S.D. To identify the differences of continuous variables between the three tissues (tumor, tumor-adjacent, and healthy tissue), we used an independent t-test for normally distributed variables and a Mann–Whitney test for non-normally distributed variables, accordingly. Likewise, the differences of continuous variables across the TNM categories were assessed accordingly. Bivariate associations between continuous variables were examined using Spearman’s rho correlation coefficient. To evaluate the possible predictive ability of AOPPs, MM9, and NF-κB for identifying colon cancer, we performed receiver operating characteristic curve (ROC) analysis.

All statistical analyses were performed by the standard IBM Statistical Package for Social Sciences-SPSS 18.0 for Windows (Chicago, IL, USA).

## 3. Results

Tumor staging with gender distribution and median age are presented in Table 1.

Stage I tumors (*n* = 3) are confined to the bowel wall, Stage II tumors (*n* = 11) have penetrated the muscularis propria, Stage III tumors (*n* = 32) have spread to involve regional lymph nodes, while Stage IV tumors (*n* = 4) have developed distant metastases. (All patients were classified as stage T4a).

A comparative table of NF-κB (%), advanced oxidation protein products (μmol/mg) and matrix metalloproteinase 9 (ng/mg) median values (minimum–maximum) in colon cancer, tumor-adjacent, and healthy tissue, accordingly, is shown in Table 2. The median values of all three markers are gradually increased from healthy tissue to tumor-adjacent and colon cancer tissue.

**Figure 1:**
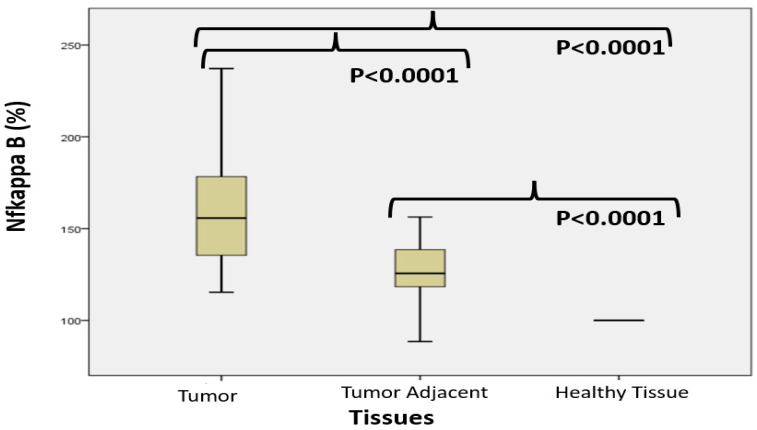
NF-κB values (%) values in colon cancer, tumor-adjacent, and healthy tissue, accordingly.

Figure 1 shows the NF-κB in colon cancer, tumor-adjacent, and healthy tissue, accordingly. The median percentage of NF-κB differed significantly among groups (Mann–Whitney test): 155.73 (115.34–251.15) in colon cancer, 125.58 (88.52–156.34) in tumor-adjacent, and 100 in healthy tissue, respectively.

**Figure 2:**
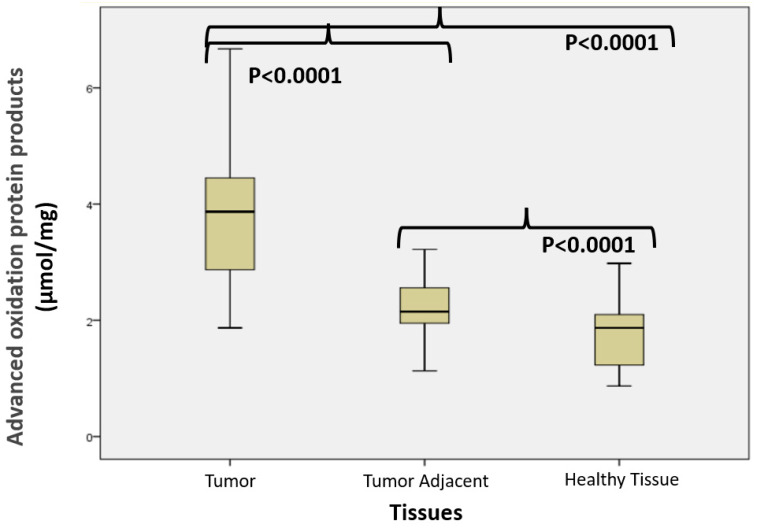
Advanced oxidation protein products (μmol/mg) in colon cancer, tumor-adjacent, and healthy tissue, accordingly.

Figure 2 shows the advanced oxidation protein products (μmol/mg) values in colon cancer, tumor-adjacent, and healthy tissue, accordingly. The median values of AOPPs differed significantly among groups (Mann–Whitney test) and were 3.83 (1.87–6.98) in colon cancer, 2.19 (1.13–4.98) in tumor-adjacent, and 1.86 (0.87–2.98) in healthy tissue, respectively.

**Figure 3:**
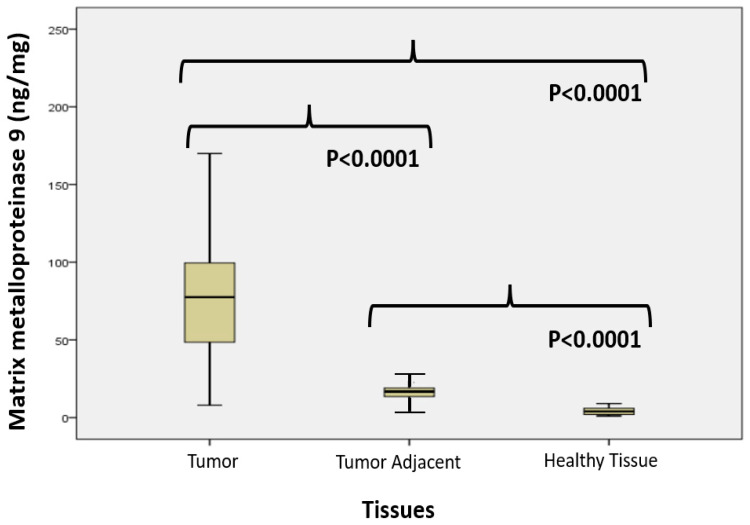
Matrix metalloproteinase 9 values (ng/mg) in colon cancer, tumor-adjacent, and healthy tissue, accordingly.

Figure 3 shows the matrix metalloproteinase 9 values (ng/mg) in colon cancer, tumor-adjacent, and healthy tissue, accordingly. The median values of MM-9 values differed significantly among groups (Mann–Whitney test) and were 77.5 (8–205) in colon cancer, 16.75 (2–32) in tumor-adjacent, and 4 (1–9) in healthy tissue, respectively.

**Figure 4:**
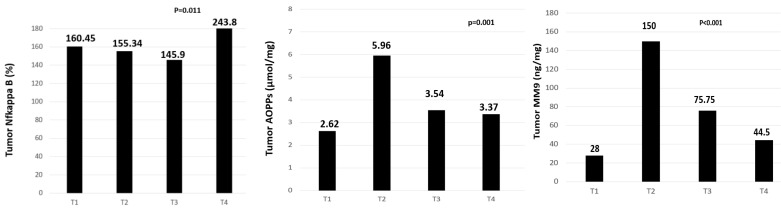
Levels of NF-κB, AOPPs, and MMP-9 in tumor tissue across different tumor stages.

We further categorized the colon cancer samples to TNM stages as stage T1, T2, T3, and T4, according to disease severity. The values of AOPPs, MM-9, and NF-κB differed significantly among TNM stages (*p* = 0.001, *p* < 0.0001, and *p* = 0.011, Mann–Whitney test, respectively). The highest levels of AOPPs and MMP-9 were observed in patients at stage II of the disease, whereas patients in stage T4 exhibited the highest levels of NF-κB (Figure 4).

**Figure 5:**
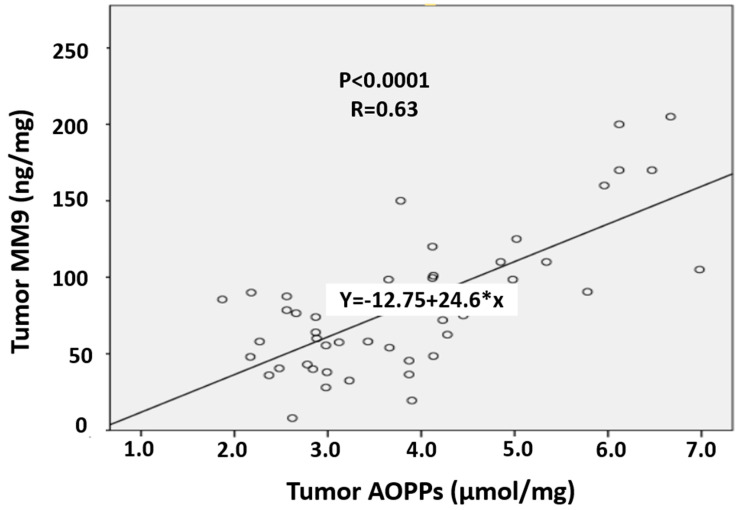
Correlation matrix between tumor AOPPs and tumor MM9 expression.

To assess the level of correlation, we performed the Spearman’s correlation test between the parameters. Tumor AOPPs were significantly correlated with tumor MMP-9 expression (*p* < 0.0001, r = 0.63, Figure 5). However, the association between tumor AOPPs and NF-κB marginally lost its significance (*p* = 0.089, r = 0.24) in the Spearman’s rho correlation matrix. There was no association between the values of AOPPs, MMP-9, and NF-κB in tumor-adjacent and healthy tissue.

**Figure 6:**
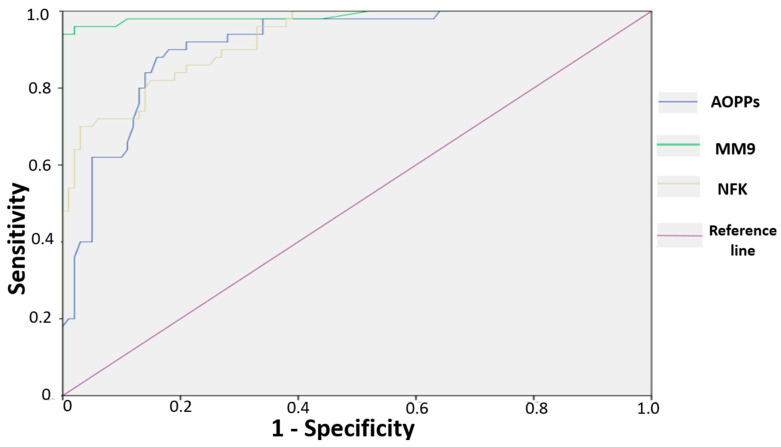
Receiver operating characteristic curve showing the performance of AOPPs, MM9, and NF-κB in predicting colon cancer.

Moreover, we aimed to investigate the predictive value of these three parameters for the diagnosis of colon cancer. The evaluation of areas under the curves (AUCs) showed that all three variables (AOPPs, MM9, and NF-κB) predicted colon cancer with particularly significantly high performance (AUC = 0.912, 95% CI = 0.87–0.96, *p* < 0.0001 for AOPPs; AUC = 0.988 95% CI = 0.97–1.00, *p* < 0.0001 for MM9; AUC = 0.927 95% CI = 0.89–0.97, *p* < 0.0001 for NF-κB), with MM9 showing the highest predictive ability (Figure 6).

## 4. Discussion

Colon cancer is a malignancy with increasing morbidity and mortality rates. During the initial phase and progression of carcinoma, the cellular mechanisms driving cancer growth continuously evolve, as multiple factors contribute to this process. Various abnormalities have been identified in oncogenes, tumor suppressor genes, mismatch repair genes, and cellular adhesion molecules [27]. However, the mechanisms underlying local progression and metastatic potential have not yet been fully elucidated, despite being key factors in tumor development. It is well established that degradation of the extracellular matrix and basement membrane is a prerequisite for tumor infiltration and metastasis. A potential mechanism could involve the activation of matrix metalloproteinases [18]. One question remains: Is inflammation a contributing factor in this process?

NF-κB, a cytoplasmic transcription factor, plays a crucial role in various biological processes, including carcinogenesis [28]. Some previous studies suggest that NF-κB is closely associated with tumorigenesis, influencing processes such as infiltration, metastasis, apoptosis, cell cycle regulation, and differentiation [29]. The results of our study indicate significantly higher NF-κB expression levels in tumor tissue compared to healthy control colon tissue and also tissue adjacent to the tumor (Figure 1). The highest NF-κB expression was observed in stage T4 (Figure 4). Due to the small number of patients with stage T4 tumors, statistical significance could not be reliably assessed for this subgroup, but the expression of the NF-κB is correlating with the tumor’s increased metastatic and proliferative potential. This finding highlights the critical role of NF-κB in promoting cell proliferation, survival, and metastasis. However, limited data are available on NF-κB activity in the tissue surrounding primary carcinoma. Our study also analyzed NF-κB expression in this adjacent, histopathologically cancer-free tissue. The results demonstrated increased NF-κB expression in all disease stages compared to distant healthy control tissue (Figure 1). The inflammatory microenvironment facilitates tumor progression through a series of dynamic and reciprocal interactions between inflammatory and tumor cells [30]. This complex interplay involves a dynamic exchange of signaling molecules and regulatory factors, ultimately establishing a microenvironment that promotes tumor growth, angiogenesis (formation of new blood vessels), and metastasis. Inflammatory cytokines, such as TNF-α, IL-6, and IL-17, released by immune cells, activate multiple signaling cascades within tumor cells [31]. In turn, tumors actively recruit and modulate various inflammatory cells, including macrophages, neutrophils, and lymphocytes, which secrete additional proinflammatory mediators that further sustain the malignant phenotype [32]. NF-κB is recognized as an important regulator in this process, promoting both the initiation and amplification of inflammation [33]. The secretion of inflammatory cytokines from tumor cells into the surrounding microenvironment further supports this hypothesis.

Activation of NF-κB is driven by the pro-oxidant state of cells, particularly the increased levels of hydrogen peroxide [34]. One of the mechanisms of NF-κB activation involves oxidative stress-mediated inactivation of phosphatases, which allows phosphorylation of I-κB and subsequent translocation of NF-κB into the nucleus. A recent study by Qiu et al. [35] demonstrated that ROS production was reduced following hyperoside administration, leading to the inhibition of NF-κB signaling pathway activation.

Our study revealed significantly elevated AOPP concentrations in tumor tissue when compared to healthy colon tissue (Figure 2). In our previous report, we demonstrated that colon cancer tissue exhibits significantly higher levels of oxidative stress markers, such as TBA-reactive substances, compared to healthy colon tissue. Moreover, the tissue adjacent to the tumor also showed increased concentrations of these oxidative products relative to control tissue. These findings suggest a potential role of oxidative stress in tumor development and local invasion [36]. The primary source of oxidants in the gastrointestinal tract is likely phagocytes, which accumulate in the mucosal secretions of patients with intestinal diseases and can generate reactive species upon activation [37]. Gut microbiota or altered mitochondrial metabolism in cancer cells may increase the ROS production in CRC. The gut microbiota plays an essential role in maintaining intestinal homeostasis and modulating host immunity and metabolism. In colorectal cancer (CRC), microbial dysbiosis is frequently observed, with both commensal and pathogenic bacteria contributing to disease progression. Certain microbial species can stimulate reactive oxygen species (ROS) production in intestinal epithelial cells, either directly or by disrupting mitochondrial function [38]. Altered mitochondrial activity in cancer cells, often associated with enhanced aerobic glycolysis (Warburg effect), further amplifies intracellular ROS levels [39]. These changes promote oxidative stress and may facilitate tumor growth and invasion. Additionally, ROS can enhance the secretion of MMPs and collagenases, as well as stimulate the production of angiogenic factors such as vascular endothelial growth factor (VEGF) and IL-8.

Notably, MMP-9 is activated by ROS, and its expression is likely regulated by oxidative stress [40]. These factors may not only promote local tumor growth but also facilitate metastasis [41]. Our results demonstrate significantly higher MMP-9 activity in tumor tissue compared to adjacent tissue and healthy control tissue (Figure 3). Our findings align with previous studies demonstrating significantly higher MMP-9 expression in colon cancer tissues compared to corresponding normal distal mucosa. Furthermore, elevated MMP-9 levels in colon cancer cells have been correlated with lymph node metastasis and Dukes’ stage [42]. These findings support the hypothesis that cancer cells induce stromal cells to degrade the ECM and basement membrane through a paracrine mechanism. Theoretically, local degradation of the basement membrane is a prerequisite for epithelial and neoplastic cells to invade normal tissues [43]. MMP-9 is produced by leukocyte granulocytes, macrophages, malignant cells, and capillary endothelial cells [44]. Another mechanism of MMP-9 activation involves NF-κB signaling. A study by Shinan Li et al. [45] suggested that ROS production by NADPH oxidase could serve as an upstream signal for MMP-9 expression. They proposed that MMP-9 activation is stimulated through NF-κB activity. However, the precise role of NF-κB in MMP-9 gene regulation remains unclear. For instance, transient overexpression of IκBα in mesangial cells only partially impaired MMP-9 upregulation, suggesting that NF-κB plays a permissive rather than a direct regulatory role in MMP-9 induction [46].

Our findings indicate that ROS may serve as an activator of NF-κB expression and that both ROS and NF-κB contribute to MMP-9 secretion. This is consistent with the study by Wu Y. et al. [47], which identified the ROS–NF-κB axis as a crucial signaling pathway in MMP-9 expression. To determine whether NF-κB activation is essential for MMP-9 expression, Bond et al. [48] utilized a recombinant adenovirus to overexpress IκBα, an endogenous NF-κB inhibitor. Overexpression of IκBα inhibited NF-κB binding and reduced both MMP-9 protein and mRNA levels, confirming the essential role of NF-κB in MMP-9 upregulation. In our samples, we tested the correlation between AOPPs, NF-κB, and MMP-9 in order to evaluate whether OS and inflammation influence MMP-9 activation. We categorized the colon cancer samples to TNM stages as stage T1, T2, T3, and T4, according to disease severity. The values of AOPPs, MMP-9, and NF-κB differed significantly among TNM stages. The association between tumor AOPPs and NF-κB marginally lost its significance, but tumor AOPPs were significantly correlated with tumor MMP-9 activity (Figure 5). Thus, based on our findings, oxidative stress is likely an important activator of MMP-9. It has been demonstrated that the intensity of oxidative stress is directly correlated with the development of colorectal cancer [49]. Avinash et al. [50] also reported an increase in AOPP levels and percent hemolysis, an indicator of membrane damage, as well as a highly significant elevation in globulin concentrations in colorectal carcinoma. The findings of that study suggest that oxidative stress and a secondary inflammatory response play critical roles in colorectal carcinoma progression. Similarly, Murlikiewicz et al. [51] reported significantly higher levels of protein oxidation in patients with colorectal cancer (CRC) compared to control subjects. Moreover, this oxidative damage increased progressively with each subsequent disease stage (according to Dukes’ classification).

There is also evidence that MMPs are overexpressed by the tumor-associated stroma [52]. Cancer cells may infiltrate and invade surrounding tissue through MMP-9–mediated degradation of the basement membrane of newly formed blood vessels, promoting tumor growth and angiogenesis through VEGF activation [53]. Additionally, MMPs contribute to the release of ECM-embedded growth factors [54], while VEGF plays a key role in vascular homeostasis by enhancing cell proliferation, stimulating neovascularization, and increasing vascular permeability [55]. However, the adjacent colon tissue, which is macroscopically and pathohistologically normal, is rarely examined. The elevated MMP-9 activity in our study (Figure 3) observed in tumor-adjacent tissue suggests its involvement in tumor invasion through the surrounding stroma. Based on our results, we conclude that signals promoting cancer spread originate from the tumor itself and influence the surrounding tissue. This peritumoral tissue may serve as a crucial site for carcinogenesis and local cancer invasion and progression, likely influenced by signaling pathways from the primary tumor. Our results suggest a critical role for MMP-9 in local invasion and tumor aggressiveness, particularly during the T2 and T3 stages (Figure 4), when the tumor breaches the basement membrane, invades local tissue, and stimulates neovascularization. These are the stages in which the tumor exhibits peak aggressiveness, locally infiltrating tissues. The results of our study show that the highest activity is in the T2 stadium of the disease. Therefore, we link the high activity in this stadium with the local invasion of cancer and the high proliferation of the cells. T3 stadium also has a high activity of MMP-9, since the tumor is making a distant metastasis and there is a need for ECM degradation. Li Zhu-Lin et al. [56] also reported the highest MMP-9 expression in stage III and attributed its reduced activity in stage T4 to MMP-9 deactivation. Furthermore, our findings indicate that peritumoral tissue is highly active in ECM degradation, reinforcing its potential role in cancer progression and invasion. Since most patients in our study were in T2 and T3 stages (Table 1), ensuring appropriate surgical margins is the most critical aspect of the operative technique. Residual peritumoral tissue could serve as a site for carcinoma recurrence and may also possess metastatic potential. The ECM regulates tumor cell growth, differentiation, and the initiation of the metastatic process while acting as a natural barrier to prevent these events. However, a transformed ECM creates a permissive microenvironment, providing a structurally altered “soil” that facilitates tumor progression and metastasis [57].

AOPPs, NF-κB, and MMP-9 also demonstrated significant predictive value in colorectal cancer. Among these, MMP-9 stood out with the highest predictive potential and could serve as an excellent biomarker not only for the diagnosis of the disease but also as a theranostic marker (Figure 5). Inhibiting this enzyme, tailored to the individual levels observed in each patient, could provide a valuable theranostic strategy in the management of CRC.

This study has several limitations. The relatively small sample size, particularly the low number of patients with T1-stage colorectal cancer, may limit the statistical power of subgroup comparisons. Larger, multi-center studies are needed to validate the predictive and theranostic potential of MMP-9 in CRC. We acknowledge that the inclusion of representative histopathological findings and immunohistochemical (IHC) analysis could provide additional value by correlating molecular alterations with tissue architecture. Furthermore, dietary habits were not systematically assessed, as the primary objective was focused on tissue-based molecular profiling. Inflammatory cytokines such as IL-12, IFN-γ, TNF-α, and IL-2, which could offer insight into the immunological background, were not measured. However, their evaluation represents a promising direction for future research investigating the link between immune activation and redox/inflammatory signaling in CRC.

Additionally, clinical trials evaluating the efficacy of MMP-9 inhibitors, tailored to individual biomarker profiles, could pave the way for personalized therapeutic strategies. A detailed analysis of the molecular changes in tumor-adjacent tissues may also contribute to optimizing surgical approaches and improving long-term patient outcomes.

## 5. Conclusions

In colorectal carcinoma tissue and the adjacent tissue, significantly increased expression of NF-κB, higher concentrations of AOPPs, and enhanced MMP-9 activity were observed. The strongest correlation was detected between AOPP levels and MMP-9 activity, indicating that oxidative stress may play a key role as a major activator of MMP-9 during the tissue invasion process. MMP-9 demonstrated the highest predictive potential, suggesting its role as a promising biomarker for both diagnostic and theranostic applications. Targeted inhibition of MMP-9, based on individualized activity levels, could offer a personalized therapeutic approach, thereby enhancing the management and clinical outcomes of patients with CRC.

Considering the observed molecular changes in the adjacent tissue, proper tumor resection with optimal surgical margins could be of great importance for improving patient prognosis and preventing recurrence and metastasis.

## Figures and Tables

**Table 1:** Tumor stages (TNM classification). m: male and f: female.

Tumor Stage	Gender		Age (Mean)
	m	f	
T1	2	1	48.3
T2	6	5	54.7
T3	18	14	62.1
T4	3	1	60.5
**In total**	** 29 **	** 21 **	** 56.4 **

**Table 2:** Comparative table of NF-κB (%), advanced oxidation protein products (μmol/mg), and matrix metalloproteinase 9 (ng/mg) median values (minimum–maximum) in colon cancer, tumor-adjacent, and healthy tissue, accordingly (Mann–Whitney test).

Tissue		All	Colon Cancer	Tumor-Adjacent	Healthy Tissue	*p* Between Tissues
	Marker					
**NF-κB (%)**		122.14 (88.52–251.15)	155.73 (115.34–251.15)	125.58 (88.52–156.34)	100	<0.001
**Advanced oxidation protein products (μmol/mg)**		2.26 (0.87–6.98)	3.83 (1.87–6.98)	2.19 (1.13–4.98)	1.86 (0.87–2.98)	<0.001
**Matrix metalloproteinase 9 (ng/mg)**		16.25 (1–205)	77.5 (8–205)	16.75 (2–32)	4 (1–9)	<0.001

## Data Availability

The original contributions presented in this study are included in the article. Further inquiries can be directed to the corresponding authors.

## Abbreviations

The following abbreviations are used in this manuscript:

CRC	Colorectal cancer
AOPPs	Advanced oxidation protein products
ROS	Reactive oxygen species
MMP-9	Matrix metalloproteinase-9
NF-κB	Nuclear transcription factor κB
